# Association Between Oral Health Status and Oral Health-Related Quality of Life in Individuals With Thyroid Dysfunction: A Cross-Sectional Study

**DOI:** 10.7759/cureus.74595

**Published:** 2024-11-27

**Authors:** Pallavi Katkam, Manjunath P Puranik, Sowmya KR

**Affiliations:** 1 Public Health Dentistry, Government Dental College and Research Institute, Bangalore, IND

**Keywords:** dental caries, oral health, oral health-related quality of life, periodontal disease, thyroid dysfunction

## Abstract

Background and aim

Any alterations in the hormonal regulation system such as thyroid dysfunction may have an impact on oral health status, which in turn may affect their oral health-related quality of life (OHRQoL). The objective of this study was to determine the association of thyroid dysfunction on oral health status and OHRQoL of subjects with thyroid dysfunction.

Materials and methods

A cross-sectional study was conducted among 150 subjects with thyroid dysfunction and 150 subjects without thyroid dysfunction aged 18-60 years from a government hospital in Bangalore city. Subjects with or without thyroid dysfunction aged 18-60 years were included in the study. Subjects who had other systemic conditions, who were pregnant or lactating, who had tobacco chewing or smoking habits, and who had undergone periodontal therapy in the past six months were excluded. OHRQoL was assessed using a pre-validated Oral Health Impact Profile-14 (OHIP-14) questionnaire. Oral health examination was done using the WHO Oral Health Assessment Form for Adults, 2013. Descriptive and inferential statistics were performed.

Results

The mean age of the subjects with and without thyroid dysfunction was 43.01±10.9 and 42±9.8, respectively, with female predominance. Most of them had a socioeconomic status of less than the lower middle class. Subjects with thyroid dysfunction showed a higher proportion of dental caries, root caries, gingival bleeding, periodontal pocket, and loss of attachment as compared to the subjects without thyroid dysfunction (p<0.05). Subjects with hyperthyroidism showed poor periodontal condition and a higher proportion of dental caries experience as compared to subjects with hypothyroidism (p>0.05). Oral health indicators showed a significant positive correlation with OHRQoL among subjects with or without thyroid dysfunction.

Conclusion

There was an association between oral health status and OHRQoL among subjects with thyroid dysfunction when compared to subjects without thyroid dysfunction. Therefore, it is necessary to inform individuals with thyroid dysfunction about the importance of preventive oral care.

## Introduction

Thyroid dysfunction is regarded as the second most common glandular disorder of the endocrine system. It was estimated that about 42 million people suffer from thyroid dysfunction in India. Decreased or increased levels of triiodothyronine (T3) and thyroxine (T4) hormones may be pathologically secreted into the blood, characterizing the conditions known as hypothyroidism and hyperthyroidism, respectively [1]. The alterations in the hormonal regulation system may have an important impact on periodontal tissues, through direct changes such as soft tissue edema, demineralization, and pathological growth of the alveolar bone, or indirect changes through enzyme defects which all modify the response of periodontal tissues to microbial pathogens [2].

Common oral findings in hypothyroidism include macroglossia, dysgeusia, compromised periodontal health, and delayed wound healing. In hyperthyroidism, oral manifestations may include increased susceptibility to dental caries, periodontal disease, enlargement of extra glandular thyroid tissue (primarily in the posterior lateral tongue), maxillary or mandibular osteoporosis, and burning mouth syndrome [3].

Oral health, not only means healthy teeth but is also integral to general health. The mouth can be the site through which microorganisms enter the susceptible host and cause various diseases/infections [1]. Oral health-related quality of life (OHRQoL) is defined as the impact of oral diseases and disorders on aspects of everyday life that a patient or person values and that are of sufficient magnitude - in terms of frequency, severity, or duration - to affect their experience and perception of their life overall [4]. Problems caused by oral diseases may cause chewing problems, less food intake with consequent weight loss, insomnia, irritability, and low self-esteem, affecting the quality of life of a person [5].

The previous literature showed that individuals suffering from thyroid dysfunction are more vulnerable and at higher risk of developing oral diseases due to bone loss and root resorption as a result of periodontal destruction [1]. There is scarce research on whether thyroid dysfunction affects oral health status and oral health-related quality of life. Therefore, the objective of this study was to determine the association of thyroid dysfunction with oral health status and oral health-related quality of life in the population. The study hypothesized that there is an association between oral health status and oral health-related quality of life in subjects with thyroid dysfunction.

## Materials and methods

A cross-sectional study was conducted among 150 thyroid dysfunction (75 hypothyroid and 75 hyperthyroid) individuals and 150 individuals without thyroid dysfunction aged 18-60 years from the outpatient department of a government hospital in Bangalore city. The study was conducted following the Helsinki Declaration [6]. A well‑defined protocol for the intended study and ethical clearance was obtained from the Institutional Ethics Committee of Government Dental College and Research Institute (#GDCRI/IEC-ACM(2)/01/2023-24) and Bangalore Medical College and Research Institute (# BMCRI/EC/11/23-24). Necessary permissions were secured from the heads of institutions/superintendents of government hospitals to conduct the study. Participants provided written informed consent after being fully informed about the study's purpose, nature, and confidentiality. The study period was from November 2023 to April 2024. This study adheres to the Strengthening the Reporting of Observational Studies in Epidemiology (STROBE) guidelines for reporting [7].

Subjects with or without thyroid dysfunction aged 18-60 years were included in the study. Subjects were excluded if they had any other systemic conditions, such as diabetes or hypertension, had a habit of tobacco chewing or smoking, had undergone periodontal therapy in the past six months, or were pregnant or lactating.

The sample size (150) was calculated using G*Power 3.1 software (Düsseldorf, Germany: Heinrich-Heine-Universität Düsseldorf) based on the data from a previous study, with an effect size of 0.3, a significance level of 0.05, and a power of 0.80 [1]. Thus, the study was conducted among 150 subjects with thyroid dysfunction and 150 subjects without thyroid dysfunction. A consecutive sampling technique was employed based on eligibility criteria.

The investigator was trained and calibrated before clinical examination. A high kappa coefficient for intra-examiner reliability was obtained (k=0.90). Data were collected using a structured proforma. General information regarding demographic profile, medical history, dental history, dietary habits, and oral hygiene practices were collected through interviews.

Oral health-related quality of life was assessed using a pre-validated Oral Health Impact Profile-14 (OHIP-14) questionnaire [8]. The Oral Health Impact Profile-14 (OHIP-14) questionnaire is composed of 14 items arranged in seven subscales (functional limitation, pain, physiological discomfort, physical disability, psychological disability, social disability, and handicap) [8]. The items were graded on a five-point Likert-type scale (0=never, 1=rarely, 2=sometimes, 3=often, and 4=very often). The overall score ranges from 0 to 56, with higher scores representing a greater impact on oral health-related quality of life (OHRQOL). Clinical examination was done by the WHO Oral Health Assessment Form for Adults, 2013, under natural light using a mouth mirror and Community Periodontal Index (CPI) probe [9]. Infection control and sterilization measures were maintained throughout the study.

Data analysis was done using SPSS software version 20.0 (Armonk, NY: IBM Corp.). The Kolmogorov- Smirnov test showed a normal distribution of data; therefore, parametric tests were used. Descriptive statistics with frequency, mean and standard deviation were computed. Chi-square test was used to determine the relationship between the variables. Differences in means between the groups were compared using an independent t-test. Subgroup analysis was done between the subjects with hyperthyroidism and hypothyroidism using chi-square test and independent t-test. Socioeconomic status was dichotomized based on median score. Duration of dental visits was categorized as less or equal to six months and more than six months. The correlation between the variables was determined using Pearson correlation test. The correlation was considered as weak (r=0.10-0.39), moderate (r=0.40-0.69), strong (r=0.70-0.89), and very strong (r=0.90-1.00) accordingly [10]. The statistical significance level was set at p<0.05.

## Results

This cross-sectional study comprised 150 subjects with thyroid dysfunction (18-34 years = 22%; 35-44 years = 28%; 45-60 years = 50%) and 150 subjects without thyroid dysfunction (18-34 years = 20.7%; 35-44 years = 39.3%; 45-60 years = 40%) with a mean age of 43.01±10.9 and 42±9.8, respectively. Most of them were female among subjects with or without thyroid dysfunction (66%; 52%) (p=0.014). The majority of them had a socioeconomic status of less than lower middle class among subjects with or without thyroid dysfunction (92%; 78.6%) (p=0.001).

About one-fourth of the subjects with or without thyroid dysfunction visited the dentist for more than six months (25%; 30) due to pain (22%; 20) (p=0.023). A higher proportion of the subjects with and without thyroid dysfunction received cleaning of teeth and filling of the tooth (16%; 21.33%), respectively, as treatment (p=0.002).

Almost all the participants with or without thyroid dysfunction used toothbrushes to clean their teeth (98%; 100%), of medium type among subjects with thyroid dysfunction and soft type among subjects without thyroid dysfunction (53.33%; 51.33%) (p=0.001). Most of the subjects with or without thyroid dysfunction cleaned their teeth in horizontal method (86.67%; 64.67%) (p<0.001), using toothpaste (93.33%; 99.33%) (p=0.006), once daily (78.67%; 68.67%) (p=0.04), with frequency of changing toothbrush in two to three months (48.67%; 74%) (p<0.001).

Subjects with thyroid dysfunction showed higher proportion of dental caries (86.6%), root caries (12.67%), gingival bleeding (66%), periodontal pocket (43.33%), and loss of attachment (19.33%) as compared to the subjects without thyroid dysfunction (67.3%; 4.67%; 44.67%; 18.67%; 6.67%), with statistically significant difference between them (p<0.05) (Table 1).

**Table 1:** Mean and proportion of subjects with or without thyroid dysfunction according to the oral health indicators. DMFT: Decayed Missing Filled Teeth; LOA: loss of attachment

Oral health indicators	Subjects with thyroid dysfunction n (%)	Subjects without thyroid dysfunction n (%)	p-Value	Chi-square value	t-Value
Dental caries					
DMFT≥1	130 (86.6)	101 (67.3)	<0.001	15.83	-
DMFT=0	20 (13.30)	49 (32.6)			
Mean±SD	4.24±3.46	2.03±1.87	<0.001	-	6.86
Root caries					
Present	19 (12.67)	7 (4.67)	0.036	6.06	-
Absent	131 (87.33)	143 (95.33)			
Mean±SD	0.26±0.79	0.10±0.47	0.036	-	2.11
Gingival bleeding					
Present	99 (66)	67 (44.67)	<0.001	13.81	-
Absent	51 (34)	83 (55.33)			
Mean±SD	4.77±3.91	2.77±3.28	<0.001	-	4.71
Periodontal pocket					
Present	65 (43.33)	28 (18.67)	<0.001	21.33	-
Absent	85 (56.67)	122 (81.33)			
Mean±SD	0.93±1.24	0.59±1.21	0.017	-	2.39
LOA					
Present	29 (19.33)	10 (6.67)	0.001	10.64	-
Absent	121 (80.67)	140 (93.33)			
Mean±SD	0.38±0.84	0.16±0.61	0.01	-	2.58

OHIP-14 scores revealed higher mean among the subjects with thyroid dysfunction (29.39±14.89) as compared with the subjects without thyroid dysfunction (21.51±10.41) in all the domains with statistically significant difference between them (p<0.001) (Table 2).

**Table 2:** Domain-wise mean of OHIP-14 scores among the subjects with or without thyroid dysfunction. OHIP: Oral Health Impact Profile

OHIP-14 domains	Subjects with thyroid dysfunction mean±SD	Subjects without thyroid dysfunction mean±SD	p-Value	t-Value
Functional limitation	4.67±2.26	2.64±1.88	0.008	8.44
Physical pain	4.29±2.34	3.33±1.76	<0.001	3.98
Psychological discomfort	4.89±2.45	3.57±2.08	0.006	5.03
Physical disability	3.66±2.26	2.91±1.75	0.002	3.2
Psychological disability	4.23±2.68	3.29±1.99	<0.001	3.42
Social disability	3.45±2.52	2.81±1.83	<0.001	2.52
Social handicap	4.21±2.12	2.95±1.62	<0.001	5.78
OHIP-14	29.39±14.89	21.51±10.41	<0.001	5.31

Subgroup analysis showed higher proportion of dental caries (88%), root caries (14.66%), gingival bleeding (72%), periodontal pocket (49.33%), and loss of attachment (22.66%) among the subjects with hyperthyroidism as compared to the subjects with hypothyroidism (85.33%; 10.66%; 60%; 37.33%; 16%), with no statistically significant difference between them (p>0.05) (Table 3). Subjects with hyperthyroidism showed higher mean OHIP-14 scores when compared with subjects with hypothyroidism with a statistically significant difference between them (p<0.05) (Table 4).

**Table 3:** Mean and proportion of subjects with hyperthyroidism and hypothyroidism according to the oral health indicators. DMFT: Decayed Missing Filled Teeth; LOA: loss of attachment

Oral health indicators	Subjects with hyperthyroidism n (%)	Subjects with hypothyroidism n (%)	p-Value	Chi-square value	t-Value
Dental caries					
DMFT≥1	66 (88)	64 (85.33)	0.631	0.231	-
DMFT=0	9 (12)	11 (14.66)			
Mean±SD	4.68±3.51	3.80±3.38	0.12	-	1.563
Root caries					
Present	11 (14.66)	8 (10.66)	0.461	0.542	-
Absent	64 (85.33)	67 (89.33)			
Mean±SD	0.28±0.76	0.24±0.83	0.76	-	0.306
Gingival bleeding					
Present	54 (72)	45 (60)	0.121	2.406	-
Absent	21 (28)	30 (40)			
Mean±SD	5.32±3.89	4.21±4.05	0.09	-	1.704
Periodontal pocket					
Present	37 (49.33)	28 (37.33)	0.138	2.199	-
Absent	38 (50.66)	47 (62.66)			
Mean±SD	1.09±1.32	0.77±1.14	0.116	-	1.581
LOA					
Present	17 (22.66)	12 (16)	0.301	1.069	-
Absent	58 (77.33)	63 (84)			
Mean±SD	0.43±0.87	0.33±0.81	0.499	-	0.679

**Table 4:** Domain-wise mean of OHIP-14 scores among the subjects with hyperthyroidism and hypothyroidism. OHIP: Oral Health Impact Profile

OHIP-14 domains	Subjects with hyperthyroidism mean±SD	Subjects with hypothyroidism mean±SD	p-Value	t-Value
Functional limitation	4.91±2.26	4.43±2.23	0.194	1.305
Physical pain	4.63±2.32	3.95±2.33	0.076	1.79
Psychological discomfort	5.27±2.34	4.52±2.52	0.062	1.88
Physical disability	3.92±2.36	3.4±2.13	0.159	1.415
Psychological disability	4.71±2.52	3.75±2.76	0.028	2.222
Social disability	3.84±2.36	3.05±2.62	0.055	1.932
Social handicap	4.64±1.97	3.77±2.19	0.012	2.547
OHIP-14	31.91±14.5	26.87±14.94	0.038	2.096

A higher mean OHIP-14 score was observed among subjects, with or without thyroid dysfunction, who had the presence of dental caries (31.71±14.44; 26.82±1.07) (p<0.001), root caries (54.68±1.53; 45.86±2.41) (p<0.001), gingival bleeding (37.71±11.14; 30.30±8.9) (p<0.001), periodontal pocket (43.82±8.51; 39.04±4.89) (p<0.001), and loss of attachment (51.93±4.21; 44.10±3.54) (p<0.001) (Table 5).

**Table 5:** Association of oral health indicators with OHRQoL among subjects with or without thyroid dysfunction. OHRQOL: oral health-related quality of life; LOA: loss of attachment; OHIP: Oral Health Impact Profile; DMFT: Decayed Missing Filled Teeth

Oral health indicators	OHIP-14	
	Subjects with thyroid dysfunction mean±SD	Subjects without thyroid dysfunction mean±SD
Dental caries		
DMFT≥1	31.71±14.44	26.82±1.07
DMFT=0	14.2±6.51	12.04±4.02
p-value	<0.001	<0.001
t-value	5.33	10.02
Root caries		
Present	54.68±1.53	45.86±2.41
Absent	25.72±12.11	20.31±9.1
p-value	<0.001	<0.001
t-value	10.38	7.39
Gingival bleeding		
Present	37.71±11.14	30.30±8.9
Absent	13.24±3.75	14.41±4.34
p-value	<0.001	<0.001
t-value	15.22	14.29
Periodontal pocket		
Present	43.82±8.51	39.04±4.89
Absent	18.35±7.33	17.48±6.37
p-value	<0.001	<0.001
t-value	19.64	16.78
LOA		
Present	51.93±4.21	44.10±3.54
Absent	23.98±10.9	19.89±8.71
p-value	<0.001	<0.001
t-value	13.53	8.71

Among subjects with thyroid dysfunction, Pearson correlation showed a significant positive, very strong correlation of gingival bleeding (0.967) (p<0.001); strong correlation of Decayed Missing Filled Teeth (DMFT) index (0.883) (p<0.001), periodontal pocket (0.870) (p<0.001), loss of attachment (LOA) (0.730) (p<0.001); and moderate correlation of root caries (0.569) (p<0.001), with OHIP-14. Regarding subjects without thyroid dysfunction, there was a strong correlation between gingival bleeding (0.855) (p<0.001), DMFT (0.885) (p<0.001), periodontal pocket (0.815) (p<0.001); moderate correlation of LOA (0.584) (p<0.001), root caries (0.508) (p<0.001), with OHIP-14 (Table 6).

**Table 6:** Correlation of oral health indicators with OHRQoL among subjects with thyroid dysfunction. *P<0.001 was considered significant. OHRQoL: oral health-related quality of life; DMFT: Decayed Missing Filled Teeth; LOA: loss of attachment; OHIP: Oral Health Impact Profile

Oral health indicators	OHIP-14 score (r-Value)
DMFT	0.883*
Root caries	0.569*
Gingival bleeding	0.967*
Periodontal pocket	0.870*
LOA	0.730*

## Discussion

The associations between periodontal diseases and systemic diseases, especially those causing hormonal disturbances, such as diabetes mellitus and thyroid dysfunction, received great attention. Thyroid dysfunction, as a hormonal disorder, may manifest in any system of the body, including the mouth. The oral cavity is adversely affected by either an excess or deficiency of these hormones [11].

Sociodemographic factors are important to oral health because they can influence oral health habits and access to care [12]. The present study included subjects aged 18-60 years, reporting a higher proportion of thyroid dysfunction among the increased age group. The current study showed female predominance for thyroid dysfunction which is in line with previous studies [1,2,13]. This is probably because thyroid dysfunction is one of the most common disorders of the endocrine system and is more prevalent in females. Also, females have seven times more chances of developing thyroid dysfunction than males [1].

Socioeconomic status acts as an important factor affecting both prevalence and prognosis, even if biological variables still play a major role in the onset and course of many illnesses [14]. The majority of the subjects in this study with thyroid dysfunction belong to a social class below the lower middle. Several mechanisms are known to link socioeconomic status with thyroid function. In lower socioeconomic groups, dietary deficiencies, health behaviors, and quality and accessibility of healthcare services can affect thyroid hormone production and metabolism.

A person's dental history is crucial, as a dentist may be the first to detect early signs of underlying systemic illnesses [3]. Most of the subjects, in this study did not visit the dentist and those who did, went primarily due to pain, generally after more than six months. There was a lack of understanding among the subjects regarding the dental visits.

Good oral hygiene practices are important for people with thyroid conditions because they can help prevent oral issues that could affect health. Subjects with thyroid dysfunction in the current study revealed inferior oral hygiene practices as compared to the subjects without thyroid dysfunction contrasting earlier studies [13].

When compared, subjects with thyroid dysfunction revealed a higher proportion (86.6%) and mean (4.24±3.46) of dental caries experience and the presence of root caries (12.67%) with significant difference between both the groups. Kshirsagar et al. and Al-Rubbaey stated similar results of increased caries experience among subjects with thyroid dysfunction [1,15]. This can be due to a reduction in salivary flow rate, associated with the reduction in buffer capacity and salivary pH, which also affects oral sugar clearance negatively, and may cause an increase in the severity of dental caries among those with thyroid dysfunction [15].

In the current study, poor periodontal health status was displayed among thyroid dysfunction subjects with significantly higher proportion and mean for gingival bleeding (66%; 4.77±3.91), periodontal pocket (43.33%; 0.93±1.24), and loss of attachment (19.33%; 0.38±0.84) when compared with the subjects without thyroid dysfunction. These results were in line with the previous studies reporting poor periodontal health status among thyroid dysfunction subjects [1,11,15]. However, one study did not show significant associations of periodontal disease with thyroid diseases [13]. It was observed that subjects with hyperthyroidism showed poor periodontal condition and a higher proportion of dental caries experience as compared to subjects with hypothyroidism, which was in line with the previous studies [2,15]. It is still unclear why there is an increase in the initiation or progression of periodontal diseases in patients with thyroid dysfunction. The reasons may include a decrease in serum levels of thyroid hormones, which can enhance periodontal destruction, increase the number of resorbing cells, and make the alveolar bone surrounding the tooth less sensitive to changes in hormonal levels [1].

Oral health is closely related to general health and people’s quality of life (QoL), through affecting their oral functions and social interactions [16]. None of the studies reported oral health-related quality of life among thyroid subjects. The current study displayed significantly higher mean OHIP-14 scores in all domains, including OHIP-14 scores among subjects with thyroid dysfunction when compared to subjects without thyroid dysfunction. Oral health indicators, such as dental caries, root caries, gingival bleeding, periodontal pockets, and loss of attachment, showed a significant association with higher mean OHIP-14 scores in subjects with thyroid dysfunction compared to those without thyroid dysfunction. This suggests that individuals with thyroid dysfunction have a lower oral health-related quality of life (OHRQoL) than those without the condition.

None of the previous studies reported a correlation between oral health indicators and OHRQoL of thyroid dysfunction subjects. In the present study, oral health indicators (dental caries, root caries, gingival bleeding, periodontal pockets, and loss of attachment) showed a significant positive correlation with OHRQoL of subjects with or without thyroid dysfunction. This implies that poor oral health in individuals is associated with lower OHRQoL.

The strength of this study lies in its evaluation of OHRQoL in individuals with thyroid dysfunction, being the first of its kind. Additionally, the clinical assessment of their oral health further contributes to the existing literature. There are a few limitations to this study. A cross-sectional design does not allow for drawing conclusions about causal relationships. Reporting and social desirability biases are inherent in any self-administered questionnaire, although the subjects were assured of the confidentiality of their data.

Future longitudinal studies should be conducted to assess the effects on oral health and discover how thyroid gland treatment can influence oral health status in subjects with thyroid dysfunction. Dental professionals should consider modifications to dental care for patients with thyroid conditions such as protection of the thyroid gland with a thyroid collar while taking x‑rays. Oral health awareness and education regarding oral and systemic effects of thyroid dysfunction should be promoted among both patients and healthcare providers. Regular, two-way communication between oral healthcare providers and endocrinologists is essential for treating patients with thyroid dysfunction. Endocrinologists need to be aware of the oral manifestations of the disease, while oral healthcare providers should stay informed about thyroid control medications to effectively manage their patient's oral health.

## Conclusions

The present study found an association between oral health status and OHRQoL among subjects with thyroid dysfunction, with poor oral health and OHRQoL as compared to the subjects without thyroid dysfunction. Effective collaboration between dentists and endocrinologists is necessary. Dentists can aid in the early detection of thyroid dysfunction through oral manifestations, while endocrinologists represent an opportunity to expand a dentist’s referral base. Together, they ensure comprehensive care, improving overall health outcomes for patients with thyroid conditions. Hence, it is necessary to develop and implement timely preventive and therapeutic strategies compatible with the patient’s ability to undergo and respond to dental care.

## References

[1] Kshirsagar MM, Dodamani AS, Karibasappa GN, Vishwakarma PY, Vathar JB, Sonawane KR (2018). Assessment of oral health status and treatment needs among individuals with thyroid dysfunction in Nashik City (Maharashtra): a cross-sectional Study. Contemp Clin Dent.

[2] Monea AM, Csinszka IA, Cosarca A, Beresescu G (2015). Oral health status in patients with thyroid disorders. Int J Sci Res.

[3] Chandna S, Bathla M (2011). Oral manifestations of thyroid disorders and its management. Indian J Endocrinol Metab.

[4] Nqcobo C, Ralephenya T, Kolisa YM, Esan T, Yengopal V (2019). Caregivers' perceptions of the oral-health-related quality of life of children with special needs in Johannesburg, South Africa. Health SA.

[5] Spanemberg JC, Cardoso JA, Slob EM, López-López J (2019). Quality of life related to oral health and its impact in adults. J Stomatol Oral Maxillofac Surg.

[6] (2013). World Medical Association Declaration of Helsinki: ethical principles for medical research involving human subjects. JAMA.

[7] Vandenbroucke JP, von Elm E, Altman DG (2007). Strengthening the Reporting of Observational Studies in Epidemiology (STROBE): explanation and elaboration. Epidemiology.

[8] Slade GD (1997). Derivation and validation of a short-form oral health impact profile. Community Dent Oral Epidemiol.

[9] (2013). Oral Health Surveys: Basic Methods. Fifth Edition. http://who.int/publications/i/item/9789241548649.

[10] Schober P, Boer C, Schwarte LA (2018). Correlation coefficients: appropriate use and interpretation. Anesth Analg.

[11] Hanau KJ, Naoom ER, Mahammed HO (2018). Cpitn in Iraqi females with thyroid dysfunction. Mustansiria Dent J.

[12] de Abreu MH, Cruz AJ, Borges-Oliveira AC, Martins RC, Mattos FF (2021). Perspectives on social and environmental determinants of oral health. Int J Environ Res Public Health.

[13] Kwon M, Jeong YJ, Kwak J, Jung KY, Baek SK (2022). Association between oral health and thyroid disorders: a population-based cross-sectional study. Oral Dis.

[14] Wagner K (2024). The effects of socioeconomic status on the prevalence and prognosis of thyroid disease. J Thyroid Disord Ther.

[15] Al-Rubbaey Y, El-Samarrai S (2009). Oral health status and dental treatment needs in relation to salivary constituents and parameters among a group of patients with thyroid dysfunction. J Baghdad College of Dentistry.

[16] Baiju RM, Peter E, Varghese NO, Sivaram R (2017). Oral health and quality of life: current concepts. J Clin Diagn Res.

